# Sex Differences in Motor Unit Behavior in Patients With Parkinson's Disease

**DOI:** 10.1111/ejn.70191

**Published:** 2025-07-07

**Authors:** Yuichi Nishikawa, Kohei Watanabe, Aleš Holobar, Tetsuya Takahashi, Noriaki Maeda, Hirofumi Maruyama, Allison Hyngstrom

**Affiliations:** ^1^ Faculty of Frontier Engineering, Institute of Science & Engineering Kanazawa University Kanazawa Japan; ^2^ Laboratory of Neuromuscular Biomechanics, School of Health and Sport Sciences Chukyo University Nagoya Japan; ^3^ Faculty of Electrical Engineering and Computer Science University of Maribor Maribor Slovenia; ^4^ Department of Neurology MNES Inc. Hiroshima Japan; ^5^ Division of Sports Rehabilitation, Graduate School of Biomedical and Health Sciences Hiroshima University Hiroshima Japan; ^6^ Department of Clinical Neuroscience and Therapeutics, Graduate School of Biomedical and Health Sciences Hiroshima University Hiroshima Japan; ^7^ Department of Physical Therapy Marquette University Milwaukee Wisconsin USA

**Keywords:** electromyography, motor unit, Parkinson's disease, sex difference

## Abstract

The aim of this study was to determine whether there are sex differences in motor unit firing behavior in patients with Parkinson's disease. Twenty‐seven patients with Parkinson's disease (females = 14 [age = 71.1 ± 6.8], males = 13 [age = 69.2 ± 10.3], Unified Parkinson's Disease Rating Scale Part III score; females = 10.8 ± 4.8, males = 11.4 ± 1.4) performed a contraction at 30% of the maximal voluntary contraction. For each participant, motor unit spike trains were decomposed from high‐density surface electromyography data recorded from bilateral vastus lateralis muscles via blind source separation algorithms. In addition to the mean discharge rates, persistent inward currents were estimated via a paired motor unit analysis. Females presented significantly greater laterality of discharge rate (*p* = 0.001) and persistent inward currents (*p* = 0.0121) than males. A significant correlation was observed between the discharge rate and the recruitment threshold on the bilateral side of males and the less‐affected side of females but not on the more‐affected side of females. These findings indicate that sex differences in motor unit behavior exist in Parkinson's disease patients. Motor unit behavior may be a sensitive and quantitative evaluation tool to highlight differences in disease presentation between males and females.

## Graphical abstract

This study utilized high‐density surface electromyography and motor unit decomposition to investigate sex differences in motor unit firing behavior in patients with Parkinson's disease. Females exhibited greater asymmetry in firing properties between more‐ and less‐affected sides, including lower estimates of persistent inward currents (Δ*F*), higher discharge rates, and increased interspike interval variability. In contrast, males demonstrated more symmetrical and stable motoneuron activity.

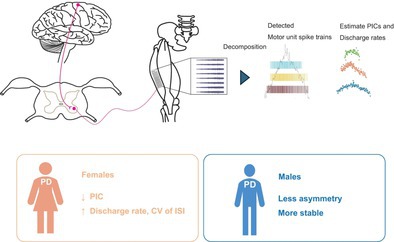

**Abbreviations**
CIs: confidence intervals
CV: coefficient of variation
EMG: electromyography
HD‐sEMG: high‐density surface electromyography
IDR: instantaneous discharge rate
ISI: interspike interval
MVC: maximal voluntary contraction
PD: Parkinson's disease
PIC: persistent inward current
PNR: pulse‐to‐noise ratio
QOL: quality of life
RT: recruitment threshold
UPDRS: Unified Parkinson's Disease Rating Scale
VL: vastus lateralis

## 1 Introduction

The incidence of Parkinson's disease (PD) is reported to be approximately 175 cases per 100,000 people, making PD one of the most common neurodegenerative diseases (Osaki et al. 2011). The motor symptoms of PD are caused by the degeneration of dopaminergic neurons within the basal ganglia. This degeneration results in impaired coordination of muscle output, leading to various motor symptoms that can profoundly affect quality of life (Cano‐de‐la‐Cuerda et al. 2010; Stevens‐Lapsley et al. 2012). Accumulating evidence indicates clear sex differences in the epidemiological and clinical features of PD. For example, females are more likely to present with tremor‐dominant symptoms and have greater motor complications, whereas males often exhibit more severe cognitive decline and bradykinesia (Georgiev et al. 2017; Picillo et al. 2017). Although males are twice as likely as females to suffer from PD (Baldereschi et al. 2000; Solla et al. 2012), females have a higher mortality rate and faster disease progression (Dahodwala et al. 2018). Furthermore, female patients with PD have been reported to respond less favorably to pharmacotherapy and to experience greater side effects from dopaminergic medications. In addition, they may derive less benefit from deep brain stimulation and report lower health‐related quality of life (QOL) scores, particularly in the physical functioning and socioemotional domains than male patients (Georgiev et al. 2017). Health‐related quality of life (QOL) is a multidimensional scale used to evaluate the impacts of disease and treatments on the lives of patients. A recent study exploring the relationships between three health‐related QOL domains (physical functioning, cognitive, and socioemotional domains) and sociodemographic variables revealed that female sex was a negative predictor of physical functioning and socioemotional health‐related QOL (Balzer‐Geldsetzer et al. 2018). Moreover, sex differences in clinical presentation have been noted, with females having a greater incidence of tremor as the initial symptom (Haaxma et al. 2007) and a higher risk of levodopa‐related motor complications due to greater postural instability (Colombo et al. 2015). In contrast, males have been identified as being at higher risk of frozen gait (Kim et al. 2018) and postural abnormalities (e.g., camptocormia) (Ou et al. 2018). Taken together, these results suggest that sex differences in voluntary force generation and motor output may exist in patients with PD. Despite the clinical implications, these properties have not been fully explored.

The recruitment of motor units and modulation of their discharge rate are mechanisms by which the central nervous system controls force production (De Luca et al. 1982). Previous research employing intramuscular electromyography (EMG) has shown that individuals with PD exhibit abnormal motor unit discharge patterns (Milner‐Brown et al. 1979; Dengler et al. 1986). A study conducted by Glendinning and Enoka revealed that individuals with PD displayed higher rates of variable and intermittent motor unit discharges at lower force recruitment thresholds (RTs) than did healthy individuals during submaximal voluntary contraction (Glendinning and Enoka 1994). Moreover, recent high‐density surface EMG (HD‐sEMG) research has shown that individuals with mild symptoms of PD have higher motor unit discharge rates for a given relative torque level than healthy individuals (Nishikawa, Watanabe, Holobar, Maeda, et al. 2021). Additionally, a notable association has been observed between neurodegeneration and the discharge rate of motor units, where individuals with more advanced neurodegeneration exhibit a higher discharge rate (Nishikawa, Watanabe, Holobar, Takahashi, et al. 2021). These findings suggest that the assessment of motor unit firing behavior could serve as a valuable method for identifying the levels of and sex differences in neurodegeneration in patients with PD.

Lower motoneuron intrinsic excitability is widely recognized to be influenced by persistent inward currents (PICs). PICs are composed of Na^+^ and Ca^2+^ currents, which increase cellular excitability by amplifying and extending synaptic inputs through the voltage‐sensitive activation of metabotropic receptors (Heckman et al. 2008). The magnitude of PICs is determined by brainstem monoaminergic input to the spinal cord. Noradrenaline and serotonin are examples of monoamines that are known to facilitate PICs and increase the gain of motoneurons in response to synaptic inputs (Johnson and Heckman 2014). Serotonin is a neurotransmitter that undergoes degeneration beginning in the initial phases of PD, resulting in low serotonin levels even in patients with mild cases (Braak et al. 2004). As serotonin levels decline, PICs may also be expected to decrease. While Madsen et al. demonstrated reduced 5‐HT receptor binding in various cortical and subcortical brain regions (Madsen et al. 2011), these findings may reflect broader serotonergic system degeneration that could extend to spinal motoneurons. Specifically, reductions in 5‐HT receptor binding were observed in regions such as the prefrontal cortex, anterior cingulate cortex, hippocampus, thalamus, and amygdala. Given that serotonin plays a key role in modulating motoneuron excitability through 5‐HT receptors located on the spinal cord, such central serotonergic deficits may contribute to the reduced PICs observed in patients with PD. Based on these findings, the hypothesis that females may exhibit lower PIC amplitudes than males is reasonable; however, to our knowledge, no previous studies have reported sex differences in the PIC amplitude in patients with PD. A study of lower limb muscles (tibialis anterior, soleus, and gastrocnemius muscles) in young healthy adults reported that the PIC is higher in females than in males (Jenz et al. 2023). However, the excitability of motoneurons is known to be affected by aging, and the PIC amplitude has been shown to be significantly reduced in older adults aged 60 and above compared with younger individuals (Hassan et al. 2021). To date, no investigations of sex differences in those over 50 years of age have been reported (Bae et al. 2008). Therefore, the effect of sex differences on elderly individuals also likely reduces the PICs. Moreover, asymmetry between limb muscles has not been studied across age groups. In patients with PD, who present with neurodegenerative asymmetries in disease characteristics, investigations of PIC asymmetry and its sex‐specific features may provide important insights into disease pathophysiology.

The aim of this study was to determine whether sex differences in abnormalities in motor unit firing behavior exist in patients with PD using HD‐sEMG. We hypothesized that PICs would be lower in females than in males and that asymmetries in PICs and discharge rates would be more pronounced in females.

## 2 Materials and Methods

### 2.1 Subjects

A total of 27 individuals with PD (14 females and 13 males) were included in this study after providing informed consent. The research methodology and methods were authorized by the Committee on Ethics in Research at Hiroshima University Hospital (approval number 76‐2) and adhered to the guidelines outlined in the Declaration of Helsinki. The criterion for enrollment was a medical diagnosis of PD. Diagnoses of other neurological disorders (Parkinson's syndrome, dementia, myositis, spinal muscular atrophy, and dystonia) and injuries to the lower limbs were the exclusion criteria. Every patient with PD was evaluated by the same neurologist using the Unified Parkinson's Disease Rating Scale (UPDRS) Part III to characterize disease severity (e.g., the more severe side was defined as the more affected side).

### 2.2 Experimental Protocols

**FIGURE 1:**
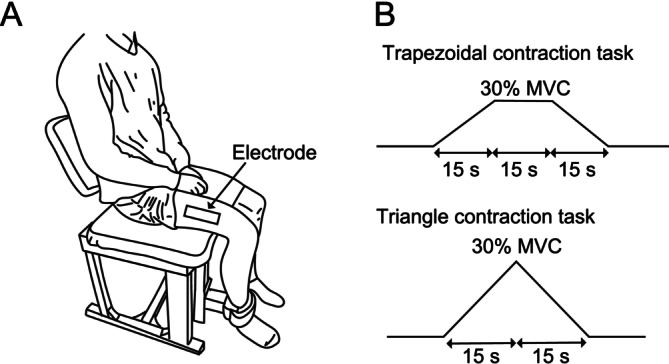
Study protocol. The participants were seated with their lower distal extremity secured in a device to measure isometric knee extension force, with high‐density surface electromyography electrodes placed on the vastus lateralis muscle (A). During the experiment, two different types of real‐time visual feedback were provided on a monitor in front of the participants (B).

The experimental procedures were executed using the same methodology as in a prior investigation (Nishikawa et al. 2022). The assessment included all participants seated in a bespoke dynamometer (TSA‐110, Takei Scientific Instruments, Niigata, Japan), with their hip and knee joints set at a 90° flexion angle (with full knee extension at 0°) (Figure 1A). The torque signals were recorded with an analog‐to‐digital converter and amplifier (Quattrocento, OT Bioelettronica, Turin, Italy), sampled at a frequency of 2048 Hz and smoothed offline with a 10‐Hz lowpass filter (fifth‐order Butterworth filter). The participants were instructed to perform a maximal isometric knee extensor contraction in two separate trials for each leg, with a 2‐min interval between each trial, preceded by a 10‐min warm‐up. During maximal voluntary contraction (MVC), the participant systematically increased the torque produced by the knee extensor muscles from zero to its highest level in 3 s and maintained this maximum torque for 2 s (Nishikawa et al. 2019). The peak torque was used as the maximal torque level to determine the submaximal target torques for the subsequent ramp‐up contraction task. Following a minimum rest interval of 10 min after the MVC measurements, all participants underwent EMG evaluations. HD‐sEMG signals were obtained in the bilateral vastus lateralis (VL) muscles of patients with PD. The participants were instructed to complete two types of submaximal contraction tasks: (1) the sustained contraction task (i.e., the ramp and hold contraction), which included ramping up to 30% of the MVC in 15 s, maintaining the contraction for 15 s, and then ramping down for 15 s (Figure 1B); and (2) the triangle task, in which the torque was accelerated 2% MVC/s to 30% MVC within 15 s and subsequently reduced at the same pace to 0% MVC (Figure 1B) (Jenz et al. 2023). The participants were provided with immediate visual feedback of their torque, which was shown on a viewing screen. While the subjects were conducting the task, verbal encouragement was given.

### 2.3 EMG Measurements

A set of 64 electrodes, each measuring 1 mm in diameter and spaced 8 mm apart (GR08MM1305, OT Bioelettronica), was used to capture HD‐sEMG signals from the VL muscle. The recording process was consistent with the methods utilized in other investigations (Watanabe et al. 2012; Nishikawa, Watanabe, Takahashi, Hosomi, et al. 2017; Nishikawa, Watanabe, Takahashi, Orita, et al. 2017). The location of electrode placement was established in prior investigations, with the center positioned midway between the head of the greater trochanter and the lateral border of the patella (Nishikawa, Watanabe, Takahashi, Orita, et al. 2017; Nishikawa et al. 2018; Nishikawa, Watanabe, Holobar, Takahashi, et al. 2021). Following the application of 80% alcohol for skin cleansing, the electrode grid was affixed to the muscle surface via a bioadhesive sheet (KIT08MM1305, OT Bioelettronica) coated with conductive paste (Elefix ZV‐181E, NIHON KOHDEN, Tokyo, Japan) (Nishikawa, Watanabe, Takahashi, Orita, et al. 2017). A ground electrode was positioned on the patellar surface. The monopolar HD‐sEMG signals were captured by a 16‐bit analog‐to‐digital converter (Quattrocento, OT Bioelettronica) with a sampling frequency of 2048 Hz. The signals were amplified with a gain of 150 and off‐line bandpass filtered with a frequency range of 10–500 Hz. HD‐sEMG and force signals were measured in temporal synchronization using OT Biolab+ software (OT Bioelettronica) at the onset of each trial. An analysis of the force and EMG data was conducted using MATLAB software (MATLAB 2021b, MathWorks GK, MA, USA).

### 2.4 Data Processing

The motor unit discharge timings were extracted from HD‐sEMG recordings via a convolutive blind source separation method (Holobar and Zazula 2004; Merletti et al. 2008; Holobar et al. 2009). The spike trains of motor units detected via the decomposition analysis were individually assessed for signal quality by an investigator (Y.N.). Poor‐quality signals, characterized by a pulse‐to‐noise ratio (PNR) below 30 dB, suggesting a motor unit identification accuracy below 90%, were eliminated (Holobar et al. 2014). Interspike intervals (ISIs) below 33.3 or over 250 ms (30 and 4 Hz, respectively) were also deleted (Holobar et al. 2009; Watanabe et al. 2016). The ISI threshold of 33.3 ms (corresponding to 30 Hz) was chosen to exclude nonphysiological or artifact‐induced high‐frequency discharges, which may arise from decomposition errors or spike misclassifications. While true doublet discharges can occur physiologically, particularly during rapid or ballistic contractions, such activity was not expected in the isometric tasks used in this study. Therefore, this threshold was applied to increase the validity of the Δ*F* estimation and ensure consistency with previous studies that used these criteria (Afsharipour et al. 2020; Hassan et al. 2021). The instantaneous discharge rate (IDR) was defined as the reciprocal of the ISI, which was calculated between two consecutive spikes and reported in pulses per second (pps). Each IDR value thus represents the discharge rate at a given moment, which is based on a single ISI (i.e., two adjacent spikes). No smoothing or moving average was applied to the IDR value used in the basic motor unit behavior analyses. The mean discharge rates of the identified motor units were determined by calculating the average discharge rate during the sustained contraction task over a period of 15 s (Figure 1B). We defined the coefficient of variation (CV) of the motor unit discharge rate as the quotient obtained by dividing the standard deviations of the motor unit discharge rates by their respective means during the same period. Here, we excluded units with motor unit discharge rates with CVs above 30%, as previously reported by Fuglevand et al. 1993 (Fuglevand et al. 1993). The motor unit RT was determined as the percentage of maximum force exerted (%MVC) at the first discharge point of each motor unit.

**FIGURE 2:**
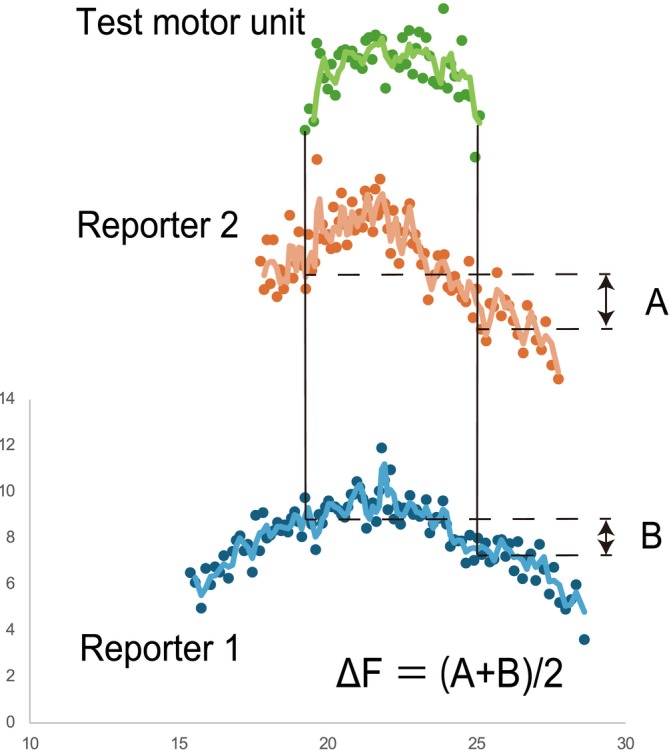
Calculation of the persistent inward currents. Example of the calculation of ∆*F* used to estimate persistent inward currents. ∆*F* is the test motor unit and is calculated as the mean lower threshold reporter unit change in the discharge rate.

Instantaneous discharge rate profiles were smoothed using support vector regression (SVR) to estimate ∆*F* (Beauchamp et al. 2022). This nonlinear regression approach was selected because of its robustness in handling noisy physiological signals, and it has been shown to outperform traditional polynomial fitting methods in the context of a motor unit discharge analysis. The smoothed discharge rate traces generated by SVR were used to quantify ∆*F* and are presented in Figure 1 for illustrative purposes. We followed established criteria for the paired motor unit analysis, including the recruitment order, rate–rate correlation, and discharge duration (Hassan et al. 2020). The quantification of the onset–offset hysteresis (i.e., ∆*F*) of a higher‐threshold (test) motor unit with respect to the discharge rate of a lower‐threshold (reporter) unit enables the estimation of the impact of PICs on motor unit discharge patterns (Afsharipour et al. 2020). Instead of establishing ∆*F* values for each test–reporter unit pair, we computed “unitwise” values, in which one ∆*F* value was assigned to each test unit by evaluating the average values derived from many reporter units during the triangle task (Figures 1B and 2). The inclusion criteria for ∆*F* values from motor unit pairs were as follows: (1) the test unit was recruited at least 1 s after the reporter unit to guarantee complete activation of the PIC (Bennett et al. 2001; Hassan et al. 2020); (2) the test–reporter unit pair showed a rate–rate correlation of at least 0.7 to ensure that motor unit pairs received a common synaptic drive (Gorassini et al. 2004; Stephenson and Maluf 2011); and (3) the reporter unit delivered a discharge rate range of at least 0.5 s per second while the test unit was active (Stephenson and Maluf 2011).

### 2.5 Statistical Analysis

All the statistical analyses were performed with Stata version 17 from Stata Corp LLC in Texas, USA. Graph creation was performed using GraphPad Prism version 10.0 from GraphPad Software Inc., California, USA. The data are presented as the means ± standard deviations. The Shapiro–Wilk test was conducted to verify the normality of the data. Separate unpaired *t* tests were conducted to detect differences in age, height, weight, disease duration, and UPDRS Part III scores between females and males. L‐DOPA and dopamine agonist usage were compared between females and males using the chi‐square test.

In this study, separate two‐way mixed‐effects models were used to analyze the CVs of the ISIs, mean discharge rates, and Δ*F*. The model included sex (females and males) and side (more‐ and less‐affected) as fixed factors, and subject as a random factor. This structure allowed us to test both the main effects and interactions while accounting for repeated measures. RT was used as a covariate in the analysis of the mean discharge rate. The mean values and 95% confidence intervals (CIs) were calculated for the discharge rate and Δ*F* under each condition. Furthermore, the Δdischarge rate and ΔΔ*F* were calculated from the difference between the more‐ and less‐affected sides for each subject. The Δdischarge rate and ΔΔ*F* were compared between females and males using an unpaired *t* test.

Pearson's correlation coefficient was calculated to determine the relationships between the CVs of the ISIs and UPDRS Part III scores, the discharge rates and UPDRS Part III scores, the Δ*F* and UPDRS Part III scores, the RT, and the discharge rates. We used locally weighted scatterplot smoothing (LOESS) regression lines to visualize the relationship between the RT and discharge rate. LOESS is a nonparametric method that estimates the local linear fit to a subset of data using a weighted least squares approach that is robust to outliers and suitable for capturing nonlinear patterns in physiological signals.

The established criterion for significance was *p* < 0.05.

## 3 Results

### 3.1 Characteristics of Participants

The characteristics of the participants are shown in Table 1. No significant differences in age (*p* = 0.572), height (*p* = 0.089), weight (*p* = 0.120), disease duration (*p* = 0.629), or UPDRS Part III score (*p* = 0.711) were observed between males and females. Males and females presented significantly lower knee extensor forces on the more‐affected side than on the less‐affected side (males; 56.88 ± 16.53 Nm vs. 65.38 ± 18.59 Nm, *p* < 0.001, 95% CI = −12.39 to −4.11 Nm; females; 48.75 ± 11.01 Nm vs. 58.32 ± 15.58 Nm, *p* = 0.036, 95% CI = −23.35 to −0.80 Nm). A significant difference was not observed between the more‐affected sides of males and females (*p* = 0.236, 95% CI = −17.50 to 4.31 Nm) and the less‐affected sides of males and females (*p* = 0.631, 95% CI = −14.04 to 8.51 Nm).

**TABLE 1:** Characteristics of participants.

	Females, *n* = 14	Males, *n* = 13	*p*
Age, years	71.1 ± 6.8	69.2 ± 10.3	*p* = 0.572
Height, cm	155.4 ± 7.4	160.9 ± 8.7	*p* = 0.089
Weight, kg	57.6 ± 9.0	62.2 ± 5.2	*p* = 0.120
Disease duration, years	2.8 ± 0.9	3.0 ± 1.4	*p* = 0.629
UPDRS Part III	10.8 ± 4.8	11.4 ± 1.4	*p* = 0.711
L‐dopa usage	12/14	12/13	*p =* 0.586
Dopamine agonist usage	4/14	3/13	*p* = 0.745

Abbreviation: UPDRS, Unified Parkinson's Disease Rating Scale.

### 3.2 Motor Unit Decomposition

A total of 353 motor units were identified in patients with PD and were considered for further analysis (females: more‐affected side = 84 motor units, less‐affected side = 99 motor units; males: more‐affected side = 76 motor units, less‐affected side = 94 motor units). On average, 4.63 motor units/subject were identified (females: 3.85 motor units/subject on the more‐affected side and 4.19 motor units/subject on the less‐affected side; males, 5.06 motor units/subject on the more‐affected side and 6.19 motor units/subject on the less‐affected side).

### 3.3 CVs of the ISIs and Discharge Rates

**FIGURE 3:**
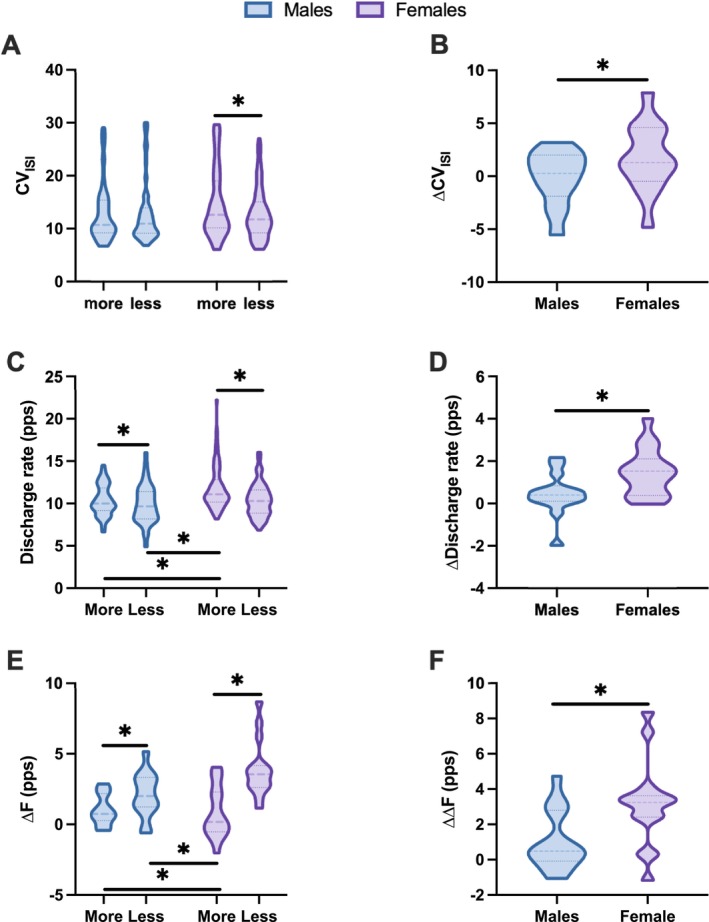
Comparison of motor unit discharge characteristics between more‐ and less‐affected sides in male and female patients with Parkinson's disease. (A) CV of ISI. (B) ∆CV of ISI (difference between more‐ and less‐affected sides). (C) Mean discharge rates. (D) ∆ discharge rate (difference between more‐ and less‐affected sides). (E) ∆*F* values indicating persistent inward currents estimates. (F) ∆∆*F* (difference between more‐ and less‐affected sides). Data are presented separately for males (left) and females (right). * *p* < 0.05. Abbreviations: CV of the ISI, coefficient of variation of the interspike interval; pps, pulse per second.

We analyzed both the CV of the interspike interval (CV of the ISI) and the mean discharge rate across participants to assess motor unit variability and output. Females showed significantly greater variability in firing patterns, as evidenced by a higher CV of the ISI on the more‐affected side than on the less‐affected side (15.14 ± 0.92 vs. 13.52 ± 0.90, *p* = 0.021, 95% CI = 0.30 to 3.70) (Figure 3A). In contrast, no significant side difference was observed in males (13.13 ± 0.99 vs. 13.46 ± 0.99, *p* = 0.319, 95% CI = −0.81 to 2.48). Notably, the between side difference in the CV (∆CV of ISI) was significantly greater in females than in males (1.69 ± 3.20 vs. −0.15 ± 2.55, *p* = 0.035, 95% CI = 0.22 to 5.49) (Figure 3B), highlighting greater asymmetry in motor unit regularity in females with PD.

We also observed that discharge rates were significantly higher on the more‐affected side than on the less‐affected side in both sexes (males: more‐affected side [10.53 ± 0.39 pps] vs. less‐affected side [9.85 ± 0.39 pps], *p* = 0.028, 95% CI = 0.07 to 1.15 pps; females: more‐affected side [11.71 ± 0.33 pps] vs. less‐affected side [10.22 ± 0.32 pps], *p* < 0.001, 95% CI = 0.89 to 2.00 pps) (Figure 3C). However, females had an overall higher discharge rate on the more‐affected side than males did, both compared with the more‐affected side (11.71 ± 0.33 pps vs. 10.53 ± 0.39 pps, *p* = 0.018, 95% CI = 0.28 to 2.88 pps) and the less‐affected side (11.71 ± 0.33 pps vs. 9.85 ± 0.39 pps, *p* = 0.001, 95% CI = 0.90 to 3.47 pps) of males (Figure 3C). Moreover, the difference in the discharge rate between sides (∆discharge rate) was significantly greater in females than in males (1.84 ± 1.12 pps vs. 0.44 ± 1.00 pps, *p* = 0.001, 95% CI = 0.59 to 2.21 pps, Figure 3D), suggesting that motor unit firing behavior may be more dysregulated in females with PD.

**FIGURE 4:**
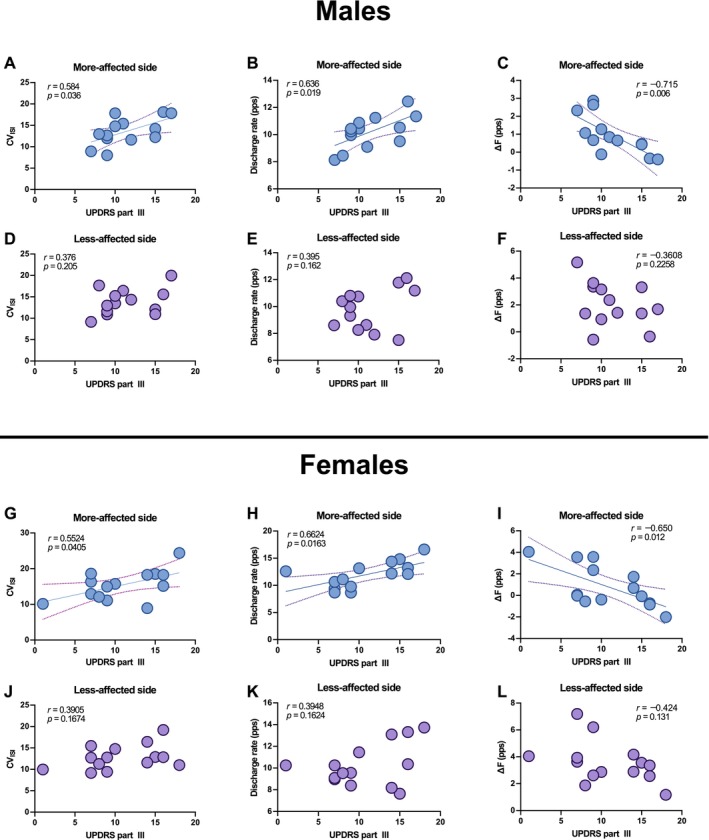
Correlations between motor unit parameters and disease severity (UPDRS Part III scores). Panels (A)–(L) show Pearson's correlations for each of the three motor unit indices—CV of the ISI, discharge rate, and Δ*F*—plotted against UPDRS Part III scores for sex (males/female) and side (more and less affected): Males—more‐affected side: (A) CV of the ISI, (B) discharge rate, and (C) Δ*F*; males—less‐affected side: (D) CV of the ISI, (E) discharge rate, and (F) Δ*F*; females—more‐affected side: (G) CV of the ISI, (H) discharge rate, and (I) Δ*F*; Females—less‐affected side: (J) CV of the ISI, (K) discharge rate, and (L) Δ*F*. Each dot represents a subject‐average value. Pearson's correlation coefficients (*r*) and *p* values are shown within each panel.

Finally, we explored the associations between these discharge characteristics and clinical severity. In both sexes, the CV of the ISI and discharge rate on the more‐affected side were significantly correlated with the UPDRS Part III score, indicating that these motor unit parameters reflect disease severity (males: CV of the ISI, *r* = 0.584, *p* = 0.036; discharge rate, *r* = 0.636, *p* = 0.019; females: CV of the ISI, *r* = 0.552, *p* = 0.041; discharge rate, *r* = 0.663, *p* = 0.016). However, such associations were not observed on the less‐affected side (males: CV of the ISI, *r* = 0.376, *p* = 0.205; discharge rate, *r* = 0.315, *p* = 0.294; females: CV of the ISI, *r* = 0.391, *p* = 0.167; discharge rate, *r* = 0.395, *p* = 0.162; Figure 4), underscoring the clinical relevance of asymmetry in neuromuscular function in patients with PD.

### 3.4 Motoneuron Intrinsic Excitability

We calculated ∆*F* as a proxy of motoneuron intrinsic excitability to estimate the PICs. Across participants, the ∆*F* values were significantly lower on the more‐affected side than on the less‐affected side in both females and males (females: 0.87 ± 1.81 pps vs. 3.91 ± 2.01 pps, *p* < 0.001, 95% CI = −4.07 to −2.06 pps; males: 1.04 ± 1.08 pps vs. 2.10 ± 1.58 pps, *p* = 0.049, 95% CI = −2.12 to −0.01 pps) (Figure 3E), which is consistent with the idea that neurodegeneration in patients with PD impairs motoneuron excitability. Importantly, compared with males, females presented a significantly lower ∆*F* on the more‐affected side (0.87 ± 1.81 pps vs. 2.10 ± 1.58 pps, *p* < 0.001, 95% CI = −4.07 to −1.73 pps, and 0.87 ± 1.81 pps vs. 1.04 ± 1.08 pps, *p* = 0.002, 95% CI = −3.01 to −0.66 pps) (Figure 3E), suggesting that excitability may be more pronounced in females with PD. We also compared the asymmetry in excitability using ∆∆*F* (the difference in ∆*F* between the more‐ and less‐affected sides), which was significantly greater in females than in males (3.14 ± 2.40 pps vs. 1.06 ± 1.69 pps, *p* = 0.0121, 95% CI = 0.50 to 3.66 pps) (Figure 3F). This sex difference in asymmetry further highlights the possibility that motoneuron dysfunction is more lateralized in females.

We explored correlations between ∆*F* and disease severity to examine the clinical relevance of these physiological differences. On the more‐affected side, ∆*F* was significantly negatively correlated with UPDRS Part III scores in both males (*r* = −0.715, *p* = 0.006) and females (*r* = −0.650, *p* = 0.012) (Figure 4). These findings indicate that reductions in PIC‐related excitability are associated with worsening motor symptoms in patients with PD. No such correlation was observed on the less‐affected side (males: *r* = −0.361, *p* = 0.226; females: *r* = −0.424, *p* = 0.131) (Figure 4), emphasizing the role of asymmetric neural degeneration in shaping motor unit behavior.

### 3.5 Correlation Between the Discharge Rate and RT

**FIGURE 5:**
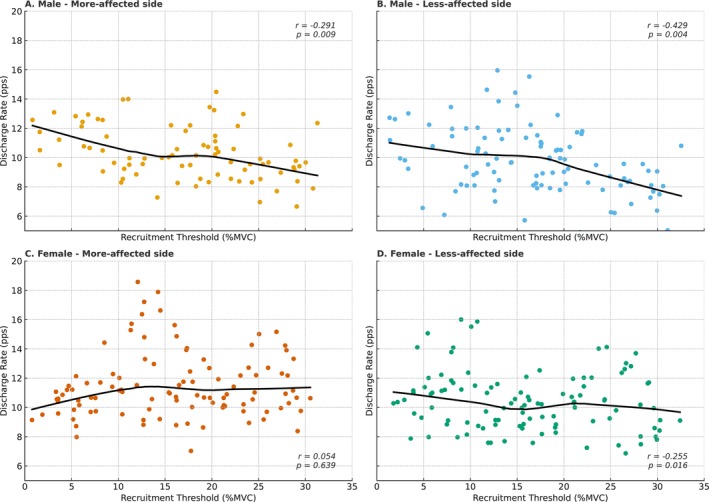
Relationships between the recruitment threshold and discharge rate across patient groups. (A) Males—more‐affected side, (B) males—less‐affected side, (C) females—more‐affected side, and (D) females—less‐affected side. Locally estimated scatterplot smoothed regression lines are shown in black. Pearson's correlation coefficients (*r*) and significance levels (*p*) are indicated in each panel.

We assessed the relationship between RTs and mean discharge rates to evaluate rate coding strategies. In males, a significant negative correlation was observed on both sides (more‐affected side: *r* = −0.291, *p* = 0.009; less‐affected side *r* = −0.429, *p* = 0.004) (Figure 5A,B), consistent with the typical onion skin pattern. In females, this negative correlation was present only on the less‐affected side (*r* = −0.255, *p* = 0.016) (Figure 5D); no such relationship was found on the more‐affected side (*r* = 0.054, *p* = 0.639) (Figure 5C). This disruption suggests impaired rate coding or recruitment order on the more‐affected side in females, despite similar clinical severity.

## 4 Discussion

This study documented sex differences in motor unit firing behavior in patients with PD using HD‐sEMG. Our primary novel results were as follows: compared with males, females presented (1) asymmetric properties of motor unit firing behavior between the more and less affected sides and (2) a greater CV of the ISI and discharge rate of motor units, a lower Δ*F*, and greater laterality of motor unit firing behavior. Furthermore, we found a significant correlation between disease severity and motoneuron properties (i.e., the more severe the symptoms were, the higher the discharge rate and CV of the ISI, and the lower the ∆*F*). This study revealed clear sex differences in motor unit firing behavior, even though the male and female participants presented comparable disease severity. These findings indicate that PD may affect neuromuscular regulation differently in males and females, independent of clinical symptom severity.

The results of this study suggest that, compared with males with PD, females with PD may have subclinical changes in motoneuron activity. Although likely multifactorial, a decrease in estradiol levels due to menopause may contribute to the differences in neuromuscular impairment in patients with PD. Estradiol is known to increase the synthesis, release, reuptake, and metabolism of dopamine (Song et al. 2019). Estradiol has also been reported to have anti‐inflammatory effects, and age‐related decreases in estradiol levels may increase systemic inflammation (Cerri et al. 2019). We previously investigated the relationship between neurodegeneration assessed by dopamine transporter single‐photon emission computed tomography and motor unit firing behavior and reported that the motor unit discharge rate was greater in patients with more advanced neurodegeneration (Nishikawa, Watanabe, Holobar, Takahashi, et al. 2021). In addition, a recent review by Piasecki et al. emphasized that menopause‐related reductions in estrogen levels contribute to motor unit remodeling, reduced discharge rates, and impaired neuromuscular control in older females (Piasecki et al. 2024). These findings collectively support the hypothesis that hormonal changes following menopause may underlie the sex differences in motor unit behavior observed in the present study. Thus, the decrease in the estradiol concentration in postmenopausal women could be an important factor in accelerating neurodegeneration and disease severity in terms of neuroprotective and anti‐inflammatory effects and may be one of the factors contributing to abnormal motoneuron activity.

This study revealed that the estimated PIC magnitudes were significantly lower on the more‐affected side than on the less‐affected side in females with PD. In males, a similar trend of reduced PICs on the more‐affected side was also observed, although the differences were not statistically significant. The values were significantly lower than those on the less‐affected side in both sexes, and a tendency for the PICs on the more‐affected side to be lower in males with PD was also observed. Lee et al. reported that PICs are proportional to the levels of the monoamine neurotransmitters noradrenaline and serotonin (Lee and Heckman 1998), and Braak et al. reported that the nucleus accumbens of patients with PD degenerates even before the onset of motor symptoms and that the production of these transmitters is reduced (Braak et al. 2004). The current findings may reflect a decrease in neurotransmitter levels resulting from the neurodegeneration that precedes motor symptoms and the greater asymmetry in females with PD, despite similar motor symptoms. Notably, the ∆*F* decreased as the discharge rate increased. PICs represent depolarizing currents that are predominantly produced by voltage‐sensitive sodium and calcium channels located in the dendrites of motoneurons (Hassan et al. 2021). PICs influence motoneuron excitability by enhancing and extending the impact of excitatory synaptic inputs. Hassan et al. reported a decrease in PICs with advancing age, alongside a reduction in the discharge rate of motor units (Hassan et al. 2021). Our findings contrast with these results, highlighting a distinct aspect of the PICs and discharge rates. This study presents the novel finding that the estimated PIC magnitude in patients with PD is low; however, numerous instances have indicated that the discharge rate of the more‐affected side appears to be elevated compared with that of healthy individuals and the less‐affected side of patients with PD (Glendinning and Enoka 1994; Nishikawa, Watanabe, Holobar, Maeda, et al. 2021; Nishikawa, Watanabe, Holobar, Takahashi, et al. 2021). The influence of motor cortex hyperexcitability may underlie this phenomenon. The compromised filtering capability of the dopamine‐depleted striatum results in a diminished inhibitory effect on subthalamic nucleus counteractions at the globus pallidus pars internus, leading to a tendency toward excessive counteractions and subsequently increased oscillatory activities in patients with PD. Consequently, motor cortex activity is elevated; however, these regions exhibit increased noise and reduced efficiency in the formation and execution of suitable motor programs (Lee et al. 2021). Based on the results of our study, the discharge rate of motor units in patients with PD may rely on the modulation of descending cortical drive to motoneuron pools to compensate for decreased PICs. Furthermore, we found that the CV of the ISI on the more‐affected side was significantly higher than that on the less‐affected side in females. The increased CV of the ISI observed in this study may reflect increased variability in synaptic input or altered central drive; however, the underlying neurophysiological mechanisms remain unclear. Although previous computational modeling studies have proposed that random fluctuations in the membrane potential or synaptic noise contribute to ISI variability (Dideriksen et al. 2012), our experimental data do not directly confirm this interpretation. Nevertheless, this asymmetry in ISI variability may support the idea of distinct sex‐specific modulation within the central nervous system, even in the presence of comparable clinical severity. In addition, we observed significant correlations between disease severity and the CV of the ISI, discharge rate, and Δ*F*. These findings are consistent with a previous study linking neurodegeneration with altered motor unit firing behavior (Nishikawa, Watanabe, Holobar, Takahashi, et al. 2021). Together, these results suggest that motoneuron properties may serve as novel physiological markers for detecting neurodegeneration, potentially even at its early stages.

Based on our findings, the typical inverse relationship between RT and discharge rate—known as the onion skin phenomenon—appeared to be preserved on both sides in males and on the less‐affected side in females with PD. However, this relationship was absent on the more‐affected side in females, suggesting impaired motor unit recruitment and rate coding. The onion skin phenomenon, originally characterized by De Luca and colleagues (De Luca and Erim 1994; De Luca and Hostage 2010), describes a pattern in which motor units recruited at lower thresholds exhibit higher discharge rates than those recruited later. Although this pattern has been supported in humans using HD‐sEMG (Del Vecchio et al. 2020), its mechanistic basis aligns with the size principle, which was first established in animal studies by Henneman (Henneman 1957). Evidence of a reverse onion skin phenomenon has also been observed at high contraction intensities (e.g., > 80% MVC) (Inglis and Gabriel 2021); however, the 30% MVC contraction used in this study is more consistent with conditions under which the conventional onion skin pattern would be expected. The disruption of this relationship in females with PD may reflect both central and peripheral mechanisms. From a central perspective, PD‐related degeneration of descending motor pathways may impair rate modulation. Peripherally, disease‐ and age‐related loss of high‐threshold motoneurons—followed by collateral reinnervation by lower‐threshold units—could reduce discharge rate variability and obscure the onion skin pattern (Lexell 1995; Piasecki et al. 2016). Similar impairments have been reported in patients with conditions such as stroke (Sauvage et al. 2006) and amyotrophic lateral sclerosis (Nishikawa et al. 2022), where central and peripheral nervous system degeneration leads to altered motor unit properties. Our study is the first to report a sex‐specific disruption of the onion skin phenomenon in patients with PD. Notably, this difference emerged despite comparable clinical symptom severity (UPDRS Part III scores) between sexes, underscoring the potential of motor unit‐level metrics to reveal subclinical sex‐specific changes in neuromuscular control.

This study has several limitations. First, in this study, motor unit firing behavior at one time point was compared in a cross‐sectional manner, and the results of this study alone cannot be used to conclude that a sex difference exists in the rate of neurodegeneration progression in females with PD. Future longitudinal studies of motor unit firing behavior and its relationship with symptom progression are needed. Second, in this study, evaluations were conducted only on the VL muscle, and whether similar results can be obtained with other muscles, especially muscles of the upper limb, is not clear. Previous studies have reported no differences in motor unit firing behavior between the dominant and nondominant leg muscles (Petrovic et al. 2022). In contrast, asymmetry in motor unit firing behavior has been observed in the upper limb (first dorsal interosseous muscle) of healthy young adults (Nishikawa et al. 2024). Therefore, caution should be exercised when extrapolating our findings to muscles of the upper extremities. Finally, in this study, neurodegeneration or hormone levels were not directly assessed. If the degree of neurodegeneration can be comprehensively evaluated by simultaneously measuring indices such as dopamine, serotonin, and estradiol levels, which are reduced by neurodegeneration in patients with PD, a more detailed understanding of PD pathophysiology can be obtained. Furthermore, although we collected data on the disease duration and UPDRS Part III scores, no significant between‐group differences were observed, and within‐group variability was limited. As a result, subgroup comparisons based on disease severity or duration were not performed, which may limit the generalizability of the findings to broader disease stages.

## 5 Conclusions

Females with PD experienced subclinical changes in motor unit firing behavior compared with males with similar physical signs and symptoms. The faster progression of clinical signs and symptoms in females with PD may be due to a significant difference in the sex‐specific nature of central neurodegeneration. Females appear to have the same physical symptoms as males do, but motor unit firing behavior may be a more sensitive evaluation tool to guide sex‐specific interventions.

## Author Contributions

**Yuichi Nishikawa:** conceptualization, data curation, formal analysis, funding acquisition, investigation, methodology, project administration, resources, software, writing – original draft. **Kohei Watanabe:** conceptualization, data curation, formal analysis, methodology, validation, visualization, writing – review and editing. **Aleš Holobar:** conceptualization, data curation, formal analysis, funding acquisition, methodology, resources, software, supervision, validation, visualization, writing – review and editing. **Tetsuya Takahashi:** data curation, methodology, validation, visualization, writing – review and editing. **Noriaki Maeda:** conceptualization, data curation, supervision, validation, visualization, writing – review and editing. **Hirofumi Maruyama:** supervision, validation, visualization, writing – review and editing. **Allison Hyngstrom:** data curation, formal analysis, methodology, supervision, validation, visualization, writing – review and editing.

## Conflicts of Interest

The authors declare no conflicts of interest.

## Peer Review

The peer review history for this article is available at https://www.webofscience.com/api/gateway/wos/peer‐review/10.1111/ejn.70191.

## Data Availability

All primary data reported here are available upon reasonable request to the corresponding author (Y.N.).
